# Artificial intelligence to differentiate asthma from COPD in medico-administrative databases

**DOI:** 10.1186/s12890-022-02144-2

**Published:** 2022-09-20

**Authors:** Hassan Joumaa, Raphaël Sigogne, Milka Maravic, Lucas Perray, Arnaud Bourdin, Nicolas Roche

**Affiliations:** 1 Department of Respiratory Medicine, Cochin Hospital, Assistance Publique - Hôpitaux de Paris (APHP), Paris, France; 2 IQVIA, La Défense, France; 3 Hôpital Lariboisière, Rhumatologie, Paris, France; 4 PhyMedExp, INSERM U1046, CNRS UMR 9214, University of Montpellier, Montpellier, France; 5 Department of Respiratory Medicine, Arnaud de Villeneuve Hospital, CHU Montpellier, Montpellier, France; 6 University Paris Descartes (EA2511), Paris, France

**Keywords:** Algorithms, COPD, Chronic obstructive pulmonary disease, Asthma, ICD code, Epidemiology, Prevalence, Healthcare administrative databases

## Abstract

**Introduction:**

Discriminating asthma from chronic obstructive pulmonary disease (COPD) using medico-administrative databases is challenging but necessary for medico-economic analyses focusing on respiratory diseases. Artificial intelligence (AI) may improve dedicated algorithms.

**Objectives:**

To assess performance of different AI-based approaches to distinguish asthmatics from COPD patients in medico-administrative databases where the clinical diagnosis is absent. An “Asthma COPD Overlap” category was defined to further test whether AI can detect complexity.

**Methods:**

This study included 178,962 patients treated by two “R03” treatment prescriptions at least from January 2016 to December 2018 and managed by either a general practitioner and/or a pulmonologist participating in a permanent longitudinal observatory of prescription in ambulatory medicine (LPD). Clinical diagnoses are available in this database and were used as gold standards to develop diagnostic rules. Three types of AI approaches were explored using data restricted to demographics and treatment dispensations: multinomial regression, gradient boosting and recurrent neural networks (RNN). The best performing model (based on metric properties) was then applied to estimate the size of asthma and COPD populations based on a database (LRx) of treatment dispensations between July, 2018 and June, 2019.

**Results:**

The best models were obtained with the boosting approach and RNN, with an overall accuracy of 68%. Performance metrics were better for asthma than COPD. Based on LRx data, the extrapolated numbers of patients treated for asthma and COPD in France were 3.7 and 1.2 million, respectively. Asthma patients were younger than COPD patients (mean, 49.9 vs. 72.1 years); COPD occurred mostly in men (68%) compared to asthma (33%).

**Conclusion:**

AI can provide models with acceptable accuracy to distinguish between asthma, ACO and COPD in medico-administrative databases where the clinical diagnosis is absent. Deep learning and machine learning (RNN) had similar performances in this regard.

**Supplementary Information:**

The online version contains supplementary material available at 10.1186/s12890-022-02144-2.

## Introduction

When they rely on adequate data, database studies can provide useful insights on disease burden as well as treatment effectiveness and safety in real-life, thereby contributing to guide decision-makers. However, such studies provide reliable disease-specific data only if the criteria applied to select patients’ populations are sufficiently robust to differentiate one disease from the other. This concern is particularly relevant in the asthma/COPD field, since these two diseases share some similarities but also exhibit many differences that have (or should have) a major impact on clinical decision-making.

Originally described under the Dutch vs British hypotheses, similarities and differences in the origins and mechanisms underlying asthma and COPD are still debated and a patient’s multi-criteria follow-up is probably the best ultimate way of discriminating the two diseases in difficult diagnostic situations. The main risk of misdiagnosis is to prevent a patient with asthma features from receiving inhaled corticosteroids: this may overtake the risks of unnecessary inhaled corticosteroids (ICS) use in COPD [1, 2], which appears frequent. In addition, identifying each entity could help identifying specific pathways and their corresponding treatment targets in the future, whereas merging the two may compromise this opportunity. A treatable trait approach has been proposed to guide treatment individualization but is still often perceived as complex to address for non-specialist clinicians and epidemiologists as well as payers.

One mission devoted to pharmaco-epidemiologists is to provide policy makers and other stakeholders with correct estimations of epidemiological trends, disease burden and resource consumption, clinical practice and size of medication-specific target populations. Databases can also be used for effectiveness and comparative effectiveness studies in real-life patients. The required precision of estimates and their specificity actually depend on the goal of the analyses. Currently, the various sources of data available for pharmaco-epidemiological studies face different strengths and weaknesses depending on whether they rely on prescriptions, international classification of diseases (ICD) coding or registries and databases specifically dedicated to asthma and COPD. The French “Système National des Données de Santé” (national health data hub) is reported as the world largest health database but diagnoses are not provided and thus need to be indirectly deduced from medication use and/or from refunded acts. In the United-Kingdom, general practitioners (GPs) databases are less exhaustive but enriched by diagnostic codes and clinical information. However, diagnoses provided by GPs sometimes change over time and do not always match with a secondary care diagnosis. Thus, at the end establishing the correct diagnosis based on a minimal amount of information collected in non-specialized clinics remains a real challenge despite the numerous available guidelines documents on asthma and COPD diagnosis and treatment [3].

Nowadays, artificial intelligence tools and computer based methods are on the rise and are gradually improving the quality of care by supporting physicians for the diagnosis as well as for the management and follow up [4–6]. Many types of algorithms have been used since the 1990s, such as artificial neural networks (ANNs), fuzzy logic (FL), Random Forests, Gradient Boosting and Logistic Regression. In the respiratory field they have been used, e.g., for the detection and classification of different types of pulmonary diseases and to predict the risk of exacerbation [7–9]. Machine learning and deep learning can both be used to build algorithms using data from medico-administrative databases [10]. Machine learning can use logistic regression models (or multinomial for multiclass classification) or boosting models (which are more adapted to assess interactions between variables but also more complex) [11]. Deep learning and especially recurrent neural networks (RNN) is adapted to longitudinal repetition of data, and therefore to sequences of health care delivery [12].

The objective of this study was to develop an algorithm able to identify asthma and COPD patients using a minimum set of data shared by medico-administrative databases. The performance of AI was tested against clinical data provided by general practitioners and pulmonologists, used as reference.

## Methods

### Study design

**Fig. 1:**
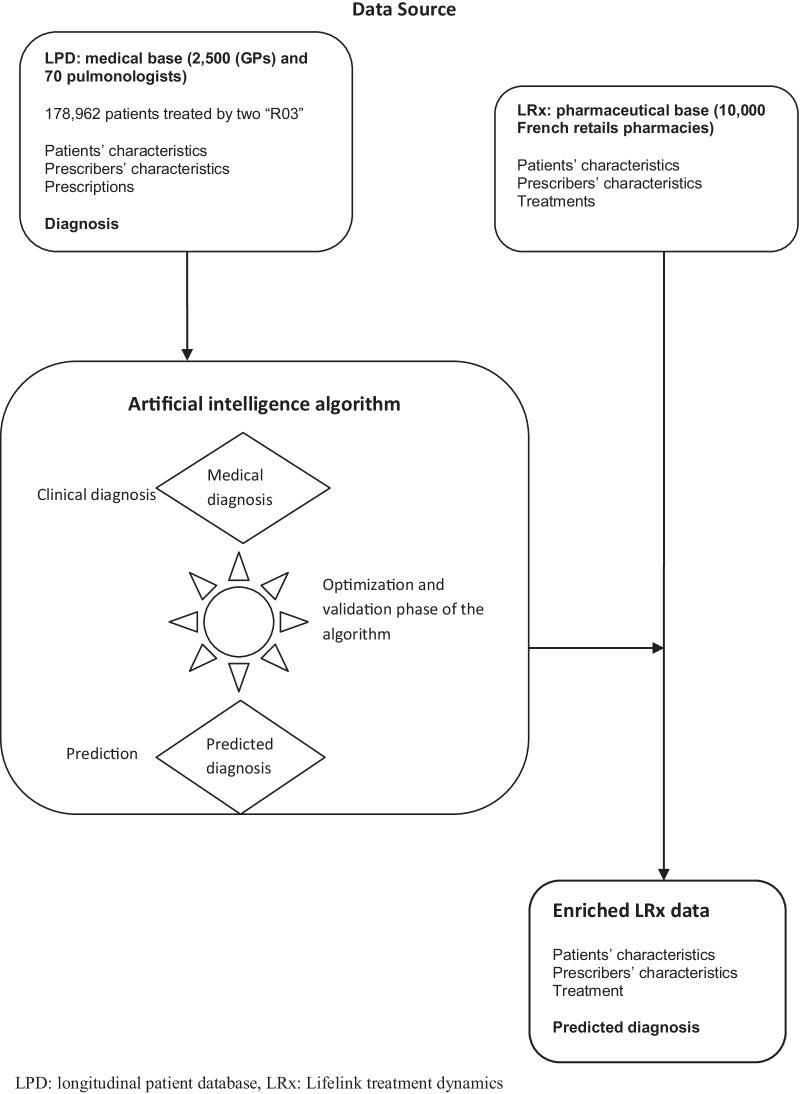
Diagram representation of the data

This was a retrospective observational database study using a medicalized and a non medicalized data source; the first represented by longitudinal patient database (LPD) and the second by lifelink treatment dynamics (LRx). A diagram illustration of the study design is represented in Fig. 1.

### Data sources

The first data source is LPD (longitudinal patient database), a database of electronic medical records from a representative computerized sample of 2,500 general physicians (GPs) and 70 pulmonologists, all office-based in private clinical practice and representative of the overall French population according to age, gender, type of practice (partial/global private activity), and geographical area of activity. Data were collected in real time during the consultation via a dedicated medical software [13]. The second source of data contains all anonymized medication dispenses prescribed in ambulatory care in a panel of 10,000 French retails pharmacies since 2012, named LRx database (Lifelink Treatment dynamics). The panel represents nearly 45% of the French retails pharmacies and is representative in terms of geographical spread in continental France and age of population coverage [14], allowing extrapolation to the overall French population. Table 1 describes the information available in each database and shared by both databases. Medical diagnoses contained in the LPD database have been used as reference to develop and validate an algorithm (using only demographics and treatment patterns) that could be subsequently applied to LRx data (where medical diagnoses are absent) to identify and differentiate patients treated for asthma and COPD in France.

**Table 1:** Information available in LPD and LRx database

	Only available in LPD database	Only available in LRx Database	Shared by both databases
Prescribers’ characteristics	Age, gender, geographical area	Geographical area	Specialty
Patients’ characteristics	Unique identification number	Unique identification number	Year of birth, gender
Visit	Date, diagnosis	–	–
Treatments’ characteristics	Number of packs, duration of the prescription and renewal	Dispensing date, dispensed volume	Prescription date, packaging, dosage, drug code (CIP)

*CIP* code identifiant de présentation (an unique code taking into account drug packaging), *LPD* longitudinal patient database, *LRx* Lifelink treatment dynamics

### LPD dataset

Selected patients were those with at least 2 R03 treatment prescriptions (i.e. treatments for obstructive airway diseases, https://www.whocc.no/) over a period of 365 consecutive days from 2016/01/01 to 2018/12/31. Each patient was assigned to one of the four following categories: patients diagnosed with both asthma and COPD: label “Both” diseases, those with asthma only, label “Asthma”, those with COPD only: label “COPD”, and those with neither asthma nor COPD diagnosis: label “other”. The dataset of each category was split into 3 parts, 80% used to train the model, 10% to validate it, and 10% to test it. We checked that data were homogeneous in each data partition thanks to a stratify split used on the patients' label.

### Models development and selection

As mentioned previously, we built diagnostic algorithms using LPD, based on the information common to both databases, i.e., patient’s profile (age, gender), prescriber’s profile (medical specialty), and prescription’s information (date, product prescribed). Importantly, models are built based on longitudinal data collected during a 2-year-follow-up. Three types of models were used: two using classic machine learning approaches, i.e. logistic regression and boosting models, and the last based on a deep learning approach, i.e. RNN. A confusion matrix was established for each model. We then selected the best model based on predictive accuracy in the test set. Based on this model, we described the demographic characteristics of the population and the top 5 treatments for each label (asthma, COPD, both, other). Additional information regarding the methodological approach is available in the supplementary material (Additional file 1: Technical Appendix).

### Prediction in the LRx database

The best model identified using LPD was applied to LRx to estimate asthma and COPD populations in this database over the period from 2018/07/01 to 2019/06/31.

### Statistical analysis

Performance metrics of best models are described using recall (i.e. sensitivity), specificity, precision (i.e. positive predictive value), negative predictive value, and F1 score. The recall is the ratio of true positives found within the population (recall = $\frac{\mathrm{tp}}{\mathrm{tp}+\mathrm{fn}}$ where $\mathrm{tp}$ is the number of true positives and $\mathrm{fn}$ is the number of false negatives). The precision is the ratio of true positives within the positive predicted population (precision = $\frac{\mathrm{tp}}{\mathrm{tp}+\mathrm{fp}}$ where $\mathrm{tp}$ is the number of true positives and $\mathrm{fp}$ is the number of false positives). F1 Score is a weighted average of both the recall and precision $\left(\mathrm{f}1\mathrm{ score }= 2 \frac{\mathrm{recall}*\mathrm{precision}}{\mathrm{recall}+\mathrm{precision}}\right).$

We performed demographic description of the predicted asthma and COPD patients in France on LRx.

## Results

### Patients

In LPD, selection criteria provided a training dataset of 178,962 patients with 1,706,130 prescriptions. Among these patients, 43%, 16%, 4%, and 37% had a diagnostic of asthma, COPD, both, and other respiratory conditions. The patients’ demographic characteristics and the top 5 treatments belonging to the R03 treatment prescriptions were described in Table 2.

**Table 2:** LPD training data: Demographics and the top 5 treatment prescription (178,962 patients)

	Asthma	COPD	Both	Other
*Demographics characteristics*				
Number of patients	76,953	28,635	7,158	66,216
Mean age (SD):	44 (25)	67 (12)	63 (16)	51 (25)
% female	56	40	53	54
*Top 5 classes of treatments (% of patients)*				
1	ICS/LABA: 68	ICS/LABA: 54	ICS/LABA: 76	ICS monotherapy: 54
2	ICS monotherapy: 39	LAMA: 47	LAMA: 47	ICS/LABA: 47
3	LTRA: 25	LABA/LAMA: 33	ICS monotherapy: 32	LTRA: 14
4	LABA: 7	LABA: 21	LTRA: 22	LAMA: 10
5	LAMA: 7	ICS monotherapy: 18	LABA/LAMA: 26	LABA: 7

*COPD* chronic obstructive pulmonary disease, *ICS* inhaled corticosteroids, *LABA* long-acting beta-2 agonist, *LAMA* long-acting muscarinic antagonist, *LPD* longitudinal patient database, *LTRA* leukotriene receptor antagonist

*ICS/LABA considered as a single drug including both product

### Model selection

**Fig. 2:**
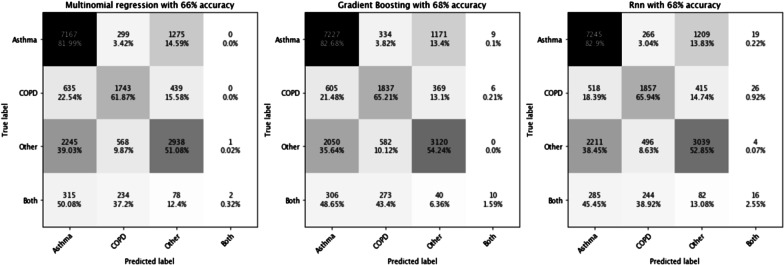
Matrices of predicted versus true cases using the different classification approaches. *COPD* chronic, obstructive pulmonary disease

Figure 2 and Table 3 showed confusion matrices of each model and performance metrics, respectively. The best models were obtained with the boosting approach and RNN, with an overall accuracy of 68%. Recall, precision and F1 score were better for asthma than COPD, while patients with other diagnoses have the worst performance metrics. Indeed, considering boosting or RNN approach, the recall was 83% and 64% for asthma and COPD, respectively; precision was 71% and 66%, respectively. As RNN did not provide any additional performance, the boosting approach was further selected in order to predict asthma and COPD populations on LRx.

**Table 3:** Performance metrics for each disease category – Boosting model/RNN

	Recall (= sensibility) (%)	Specificity (%)	Precision (= positive predictive value) (%)	Negative predictive value (%)	F1 score (%)
*Boosting model*					
Asthma	83	63	71	77	76
COPD	65	90	61	90	63
Other	54	76	66	65	60
*RNN model*					
Asthma	83	62	71	77	76
COPD	65	91	66	92	65
Other	64	84	53	77	58

*COPD* chronic obstructive pulmonary disease, *RNN* recurrent neural network

### Predicted asthma and COPD populations (LRx)

Based on LRx data, the extrapolated numbers of patients treated for asthma and COPD in France were 3.7 and 1.2 million, respectively. Patients classified as asthma were younger than COPD patients, 49.6 (25.3) years vs 72.1 (11.7) years. A male predominance was observed in COPD (68%), while men represented only 33% of patients with asthma. The number of patients considered as having both diseases was 0.4 million (mean age 69.9 (14.2), 52% male).

## Discussion

The purpose of the present project was to explore whether artificial intelligence applied to a minimal medico-administrative dataset limited to demographics and treatments could provide accurate identification of patients with asthma and COPD. First, three artificial intelligence tools (multinomial regression, gradient boosting and recurrent neural networks) were applied to a medicalized database (LPD), as a reference, to develop and test diagnostic models with the clinical diagnosis as a gold standard. The overall accuracy was similar (68%) for all models, suggesting that deep learning does not perform better than machine learning for that purpose. Second, applying the best model to a large representative non-medicalized database (LRx), allowed predicting the number of patients who receive treatments for either asthma, COPD, or both in the whole French population.

The differential diagnosis between asthma and COPD is made difficult by the lack of a single test individually allowing to differentiate these two diseases reliably. More specifically, lung function criteria (bronchial hyperreactivity for asthma, not fully reversible airflow limitation for COPD) are not sufficiently discriminative. Consequently, the diagnosis relies on a combination of clinical and lung function criteria. Among these, the clinical history plays a major role, although most questionnaires developed to date do not perform fully satisfactorily [15, 16]. Complicating diagnostic challenges even more, asthma and COPD can coexist. In 2015, the strategy updates of the Global Initiatives for COPD (GOLD) and asthma (GINA) even identified the “asthma COPD overlap”, a group of patients characterized by persistent airflow limitation with common features of both asthma and COPD like a history of allergy, exposure to tobacco smoking or air pollution and persistent respiratory symptoms with noticeable variability [17, 18]. In their most recent updates, GOLD and GINA don’t put forward anymore the use of the term “ACO”, instead stressing that asthma and COPD are different disorders even if they may share common features and be associated [2, 19, 20].

Asthma and COPD are considered by some authors as two extremes of a single continuum [21, 22], along which patients could be rather characterized using treatable traits than disease labels. From a clinical perspective, differentiating asthma from COPD has long been [23] and remains for now crucial considering the differences between these two conditions in terms of pathogenesis, natural history, prognosis and therapeutic targets [24]. Differentiating the two conditions in medico-administrative databases is therefore of interest, e.g., to estimate the prevalence of physician-diagnosed and treated asthma/COPD and the corresponding health care costs, as well as to describe and follow over time real-life clinical care for these two conditions. Such information can prove valuable to clinicians, health policy makers, health coverage systems and insurance companies, and to understand the impact of such diseases on the population.

Unfortunately, detailed clinical data is not available in most medico-administrative databases to differentiate these two diseases. An accuracy of 68% can be considered as acceptable for a tool designed to differentiate between asthma and COPD without entering in all the complexity of a medical file or patient’s diagnostic data. As such, this algorithm could be utilized as a surrogate to ICD codes when they are not available to identify asthma and COPD patients [25].

Extrapolation allowed to estimate the prevalence and demographics of asthma and COPD in the general population, with figures consistent with previous epidemiological studies using reference methods [26–29]. Our study found gender-related heterogeneity in the prevalence of COPD, asthma and both, which is also consistent with previous publications [30, 31] and could be explained by gender-related differences in the prevalence of cigarette smoking and exposure to other environmental risk factors, as well as in susceptibility [32].

Since there is a great overlap regarding recommended pharmacological treatments between asthma and COPD, it is not surprising that the medications included in Table 2 retrieved from LRx are quite similar for asthma, COPD or both. As expected, only long-acting bronchodilators without inhaled corticosteroids ICS (long-acting beta-agonists (LABA) and long-acting muscarinic antagonists (LAMA), LABA/LAMA) proved more specific for COPD, whereas leukotriene receptor antagonists (LTRA) are used only for asthma management.

Our results are consistent with those of Riccardo Di Domenicantonio et al. [35] who performed a systematic review of case identification algorithms based on the Italian health care administrative database for asthma and COPD [35]. They found that age class and chronic treatment were the main disease-specific traits that emerged from the algorithms with a lower accuracy of algorithms based on drug prescriptions for COPD patients [35]. However, validation of these algorithms for asthma was limited and provided highly variable results, while no algorithm was clearly validated for COPD. Gothe et al. [25], in another systematic review, also found that pharmacotherapy data is the most reliable and richest source of information available to identify COPD patient in an outpatient setting when ICD codes are unavailable [25]. In the validation study of healthcare administrative database algorithms to identify COPD published by Gershon et al., age was also an important criterion [36].

In several studies, the performance of AI and machine learning for the diagnosis of asthma and COPD was higher than in our study. This difference can be easily explained by the content of the databases used: our purpose was to develop an algorithm applicable to databases with no clinical data or diagnostic label. Conversely, studies that found better performance used more extensive data such as: results of spirometry, smoking status, physical examination and imaging [6, 33, 34]. For instance, Spathis and Valmos found 97.7 per cent diagnostic precision using random forest in COPD, relying on multiple elements such as smoking, age and spirometric data (FEV1 and FVC) [34].

Our results show that deep learning did not perform better than machine learning to build diagnostic algorithms. This represents important information for future medico-economic database studies in airway diseases. Indeed, RNN is usually adapted to longitudinal repetition of data, and sequences of health care delivery [37]. However, in our study, this approach did not bring additional value, underlining that the information used for the classical machine learning approach was enough to discriminate asthma from COPD. This could be explained by a training based on a dataset limited to outpatient care provided by a GP only or a pulmonologist only. Indeed, RNN could provide additional value when all the sequences of health care delivery are taken into consideration, but this remains to be further tested.

Our study has several strengths that contribute to its originality. Firstly, with only few data (i.e., sex, age and patients’ medications), we were able to develop an algorithm with an acceptable accuracy for correct identification of asthma and COPD patients. In addition, the size and representation of the LRx database confer generalizability to the present results.

The study also faces several limitations. The relatively high proportion of patients receiving R03 treatment who are not classified by physicians illustrates the diagnostic difficulties and potentially decreases the relevance of our gold standard population. This observation also needs to be put in the perspective of the marked under-diagnosis of COPD and of the lack of individual verification of medical files. In addition, models trained using asthma only or COPD only patients could have a better accuracy (i.e. > 90%, data not shown), but the challenge of overtraining needs to be questioned regarding the reality of clinical practice. The arbitrarily-defined cutoff of 2 R03 prescriptions to identify the population used to develop the algorithm could also be questioned. A sensitivity analysis was performed with cutoffs of 3 or 4 R03 prescriptions; this did not have any impact on the confusion matrix whatever the method used (data not shown). As in many health services research, case verification using chart abstraction was used to validate case definitions of asthma, COPD or both [36]. Knowing that most charts came from GP’s, the validity of our gold standard can be questioned [38]. However, in this context, the gold standard needs to be a sequence of validation processes based on history and medical course. Indeed, both our gold standard and algorithm were not based only on a single medical visit and prescription, but on a continuum of follow up over 2 years.

Moreover,LPD database describes only the patient trajectory seen by a general practitioner or a pulmonologist while LRx database describes the overall management whatever the physician and type of clinical setting. Although this could introduce some heterogeneity, it does not decrease the value of results.

Finally, our algorithm was not tested against an expert clinical diagnosis. The purpose of real-life studies performed using such databases can be to analyze data from patients “treated as” asthma or COPD, or to consider patients with a confirmed expert diagnosis of asthma or COPD. Our results apply only to the first of these types of populations, and additional studies are needed to explore their relevance in patients with a “gold standard” diagnosis.

In conclusion, this database study showed that an algorithm with acceptable accuracy can be developed to identify asthma and COPD in medico-administrative databases from which the medical diagnosis is absent. Deep learning and machine learning had similar performances in this regard. Applied to such databases, the algorithm could prove useful to estimate the burden of these diseases and to analyze clinical practice over time. Further studies are required to test the model in other populations and refine the diagnostic criteria proposed here.

## Supplementary Information

**Additional file 1.** Technical Appendix.

## Data Availability

The raw data that support the findings of this study are available from IQVIA France but restrictions apply to the availability of these data, which were used under authorization for the current study, and so are not publicly available. Data are however available from the authors upon reasonable request and with permission of IQVIA France.
