# Supplementary material for: Artificial intelligence to differentiate asthma from COPD in medico-administrative databases
Source: BMC Pulm Med. 2022 Sep 20;22:357. doi: 10.1186/s12890-022-02144-2 (PMC9487098; doi:10.1186/s12890-022-02144-2)
Supplement: Supplementary file 1 — Additional file 1. Technical Appendix. [file 12890_2022_2144_MOESM1_ESM.docx]

**Technical appendix**

1. **Introduction**

Two IQVIA databases were used for this study:

- The LPD (Longitudinal Patient database) database corresponding to electronic medical records containing physicians’ prescriptions and the associated diagnosis.
- The LRx (Lifelink Treatment dynamics) database containing the information on medications purchased in pharmacies with no diagnosis available.

Using the medicalized LPD database, we developed an algorithm that would assign a diagnosis to a patient based on the drugs’ purchased in pharmacies (i.e., given his LRx entries). This is done by *transfer learning*, i.e., by training the algorithm using the LPD data with a supervised approach and using it on LRx data. We used the longitudinal format of the data to perform the classification, using the sequence of physicians’ prescriptions. Two distinct approaches are presented in this document: a common Machine Learning approaches and a Deep Learning approaches. Throughout this document, we discuss the predictive approaches, with their respective pros and cons.

# **Featuring**

First, we proceeded with the feature engineering of our model using the common information available in both databases, i.e.: patients' profile, physicians' profile and all the information about the prescription. We distinguished two types of features: the static features (patient’s gender, prescriber’s specialty) and the longitudinal features (products prescribed along time, time delta between prescriptions).

The static features were extracted from the patient’s and physician’s profile and are the following:

- Patient’s gender (binary feature);
- Patient’s age during the period of analysis (either continuous or bucketed);
- Specialty of the prescriber, which is one-hot encoded.

The longitudinal features were extracted from the prescriptions observed over time. The features were constructed from the sequence of prescription information:

- Sequence and frequency of the prescribed medication. Each product information used is its molecular composition, dosage and format (e.g. paracetamol 500mg capsule);
- Date of prescription (e.g. month of the year);
- Frequency of prescription.

All those features were adapted in the format most suited for the model considered (e.g. the age is given as a bucket of binary features for linear models and as a continuous variable for random forests or boosting classifiers).

# **Classic machine learning modeling**

The first modelling approach used common machine learning algorithms. As a direct use of the longitudinal format of the data cannot be used, we recreated temporal information on the various considered features mentioned above and based on their respective frequency.

Here, we present different machine learning models using the same kind of features, from logistic regression to gradient boosting classifiers, and discuss their specific advantages:

- The most basic model is the **logistic regression** (or multinomial for multiclass classification). As a linear model, it is fast to train and easy to understand, especially in case of binary classification. Indeed, we can easily identify and quantify the importance of the features for the classification. However, due to its linearity, it cannot capture interaction between variables, unless feeding the model with features specifically containing such information.
- More advanced models with best performances among common machine learning approaches are **boosting models**. Slower to train, they will often deliver better performances compared to the logistic/multinomial regression. It can also understand the interaction between variables, even though feeding it with features containing this information could also help. However, understanding and interpreting those models is harder compared to the logistic regression. It is of interest to try multiple models to evaluate the problem’s complexity (e.g. whether it is linear or more complex).

# **Deep learning modeling**

Recurrent neural networks are neural networks with at least one recurrent layer, such as gated recurrent unit (GRU) and long short-term memory (LSTM) that we used in our experiments as described below. They allow dealing with inputs (and outputs) that are in the form of sequences (also called longitudinal data) such as time series, text (sequences of words), etc. Here, the input sequences are the sequences of prescriptions of the patients within the considered period of analysis.

One of the main difficulties with deep neural networks is to find out a relevant architecture (structure of the network) for the considered prediction task. In the classification problem considered here, we had two types of inputs for patients: sequences of prescriptions and static features, such as the gender and the medical specialty of the prescriber. So, we designed a neural network that can handle both types of inputs.

Below, we describe the architecture of such a network, and explain how to construct features that can be fed to this network. Then, we explain how to train this kind of neural network.

#### **Network architecture**

A neural network is defined by its architecture, which typically stacks a list of layers. As explained above, we wanted to handle inputs of two types: sequences (longitudinal features) and static features.

The longitudinal features corresponded to the set of products in a prescription; the time delta with the previous prescription; and the month of the year of the prescription. A product in a prescription is described by a code, among a list of 5000 codes. Such codes have no physical meaning and cannot therefore be used directly as inputs of a recurrent neural net (in the same way as words in a sentence are). Therefore, we had to “embed” these codes, replacing each of them by an “embedding” vector that was learned together with all the weights of the neural network. This step is called “embedding”. It is a well-understood technique, first used in the field of natural language processing (for words or documents), but also of common use with medical codes, such as codes identifying drugs or diagnosis. Note that the time delta with the previous prescription, and the month of the year did not require embedding but had to be scaled before being fed to a recurrent layer.

Therefore, the architecture of our neural network stacked the following types of layers:

1. An **embedding layer** for the codes of the products appearing in each prescription as well as the other longitudinal information, that could be fed to a recurrent layer;
2. A **self-attention layer** used to wisely aggregate the embedded vectors for each prescription;
3. A **recurrent layer**, such as a LSTM or GRU layer. It received as inputs a concatenation of the embedded product codes and the other longitudinal features. At this stage, we could use a single layer or stack several of them.
4. A **dense or classification layer** which uses the output of the recurrent layer, as well as the static features, in order to predict the label.
   1. **Embedding layer**

The first layer of the network is an embedding layer. We used this layer both on the codes of the products and the other longitudinal data. For example, it transformed the code of a product into a vector in RdEp, where dEp stands for the dimension of the embeddings. This hyper-parameter must be specified in advance and we provide results for different values below. This embedding can be understood also as a form of dimensionality reduction. Indeed, a standard encoding of these codes is the one-hot encoding, where these codes are associated with dummy binary variables. An expected advantage with vectors of embeddings is that similar products should end up with vector embeddings that are close to each other.

Once the products are embedded, we had to build an embedding for the prescriptions. Indeed, each time step corresponds to a prescription, and not to a single product. This means that at each time step, we had a list (of variable length) of products. The next layer describes a method to aggregate those embedding vectors efficiently.

- 1. **Self-attention layer**

Aggregating or fusing embedding vectors is a widely used technique, especially with the modeling of longitudinal data. For example, in natural language processing (NLP), a common thing is to fuse all the embedded word vectors within a sentence. In our case, we wanted to aggregate all the embedded product vectors within a prescription. One of the simplest aggregation strategies is the average pooling computing the average of the embedded products vectors for each dimension. Another one is the maximum pooling which keeps the maximum value for each dimension. However, both those strategies have weaknesses. Indeed, the average pooling assumes that all the products within the prescriptions have the same influence for the prediction, while the maximum pooling strategy only takes into matter the maximum value, ignoring the contribution of the other products. For our network, we introduced the use of a self-attention layer in order to fuse all the products within a prescription according to their respective importance for the prediction.

After having aggregated the embedded vectors for each consultation, the output of this step was that for each patient, we had a sequence of his prescription, his prescription being a vector of size Nproduct embedding representing the products prescribed.

- 1. **Recurrent layers**

The vectors of embeddings obtained from the self-attention layer, at each time step, were concatenated with the outputs of two other embedding layers: the embedded month of year and the embedded time delta with the previous time step. Therefore, after this concatenation, we had for each patient, at each time step, a vector of size dE that describes its prescription, with dE = dEp + dEm + dEdt, dEp being the products embedding dimension, dEm being the months embedding dimension and dEdt the time deltas embedding dimension. A recurrent layer takes as input this sequence, of shape nP ×dE, where nP is the number of prescriptions observed for the patient.

Several recurrent layers can be used; the most popular from literature are GRU and LSTM layers. GRU layers are simpler and usually faster to train while being close in terms of performance to LSTM layers. LSTM layers are more complex, but usually exhibit robust performances. Both types of recurrent layers are tested in the experiments described below.

For both types of layers, we had to choose a number of hidden cells dH, which corresponds to the number of hidden variables used at each time step. We could also stack several recurrent layers: this is computationally more involved but leads to deeper models with hopefully a better predictive power. We considered a variable number of stacked recurrent layers in our experiments. Note that, for each patient, the output of this layer (or layers) is always a vector of size dH.

- 1. **Classification layer**

The layers described above used only longitudinal features. In the classification layer of the network, we considered as input the concatenation of static features, such as the age and gender of the patient, together with the output of size dH of the recurrent layer described above. The input of this classification layer was therefore, for each patient, a vector of size dH + nS, where nS is the number of considered static features. To perform classification, it is once again possible to stack several dense layers. Each dense layer is characterized by its width (size of the outputs) and its activation function, usually chosen as the *relu* or *tanh*.

For the last layer, whose outputs correspond to the classification scores (probability of each class), the width of the layer must be K if we are considering classification with K class (let us recall that K = 4 for the asthma versus COPD classification problem). The activation function for this last layer is taken as the Softmax function, in order to produce probability scores, whenever K > 2, while it is taken as the sigmoid whenever K = 2.

In **Figure S1**, we illustrated the architecture of such network.

**Figure S1: The architecture of the neural network**


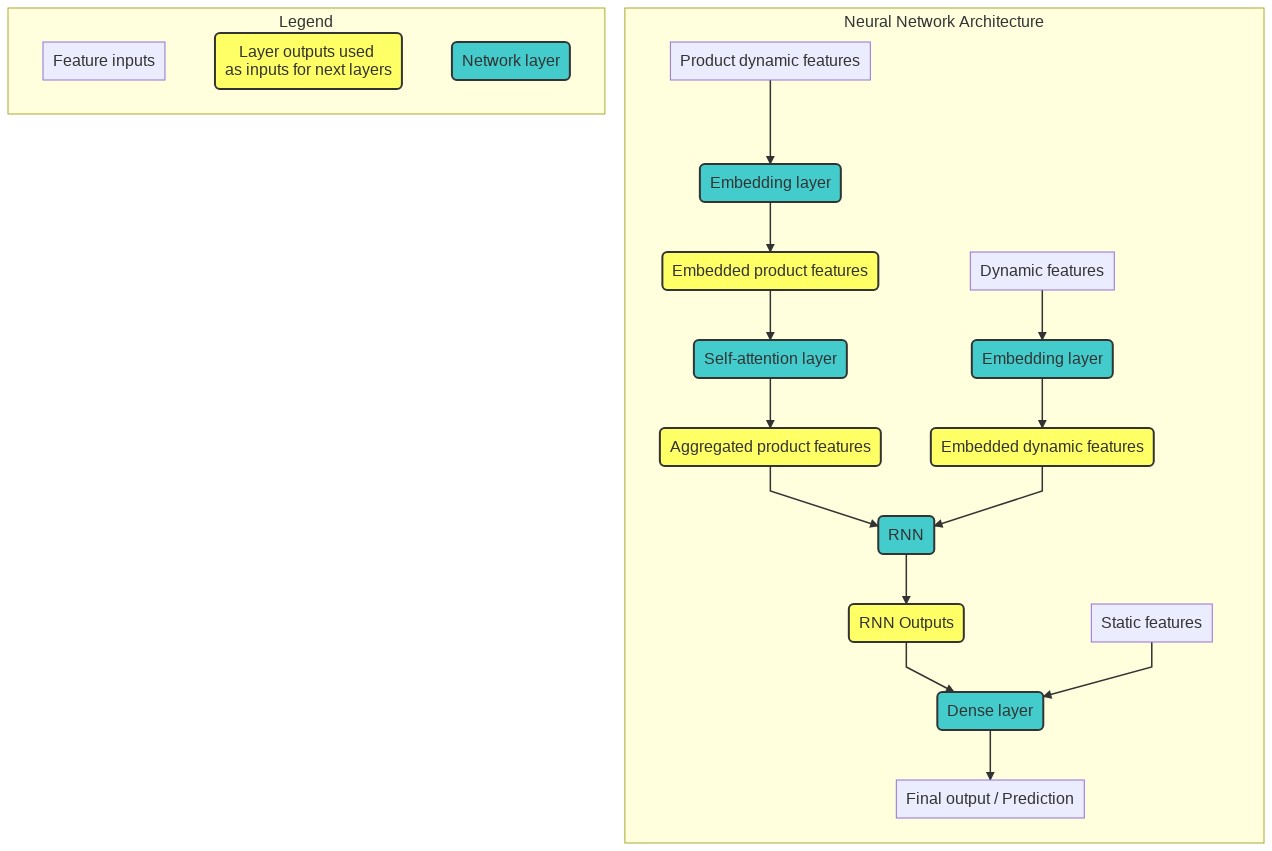


#### **Modeling**

The architecture of the neural network model described above depends on several parameters, usually called “hyper-parameters”. These hyper-parameters must be chosen with care, in order to obtain the best predictive performance. In **Table S1,** we list these hyper-parameters, recall their definition, and provide the corresponding predictive performance on the test dataset. These hyper-parameters can be tuned using cross-validation, via random grid search or more advanced strategies, such as hyper-optimization or Gaussian process techniques.

| **Table S1. Neural network parameters** | | |
| --- | --- | --- |
| **Network part** | **Parameters** | **Description** |
| Embedding layer | dE | Dimension of the embedding vectors to create |
| Embedding layer | λE | The coefficient to use for embedded vector penalization |
| Self-attention layer | K | The number of heads i.e. of self-attention function to generate |
| Output layer | dH | Either Softmax for multiclass or sigmoid for multilabel |
| General parameter | η | The learning rate of the network |
| General parameter | Npasses | The number of times we train over the dataset |
| General parameter | B | The number of patients we feed at each iteration |

GRU: gated recurrent unit, LSTM: long short-term memory
